# A comprehensive analysis of human gut microbial biosynthesis gene clusters unveiling the dominant role of *Paenibacillus*

**DOI:** 10.1128/msystems.00610-25

**Published:** 2025-06-09

**Authors:** Han Gao, Daohua Zhuang, Hong Zhou, Qian Su, Xintong Hu, Yuan Wang, Wandong Bao, Lei Zhu

**Affiliations:** 1 Cancer Research Institute & Cancer Biotherapy Center, Yunnan Cancer Hospital, The Third Affiliated Hospital of Kunming Medical University, Peking University Cancer Hospital Yunnan 531840, Kunming, Yunnan, China; 2 State Key Laboratory of Genetic Resources and Evolution, Kunming Institute of Zoology, Chinese Academy of Sciences 53028 , Kunming, Yunnan, China; 3 State Key Laboratory for Conservation and Utilization of Bio-Resources in Yunnan, School of Life Sciences, Yunnan University 12635, Kunming, Yunnan, China; Johns Hopkins Bloomberg School of Public Health, Baltimore, Maryland, USA

**Keywords:** human gut microbiota, secondary metabolite, biosynthesis gene cluster, *Paenibacillus*, polyketide synthase, non-ribosomal peptide synthetase

## Abstract

The secondary metabolites produced by the gut microbiota serve as crucial signaling molecules and substrates for gastrointestinal metabolic reactions, thereby playing a pivotal role in human physiological and pathological processes. In this study, we explore the complex symbiotic relationship between the gut microbiota and the human host by systematically annotating the biosynthetic gene clusters (BGCs) across 4,744 human gut microbiota genomes, sourced from the Unified Human Gastrointestinal Genome database. Our comprehensive analysis compares the differential biosynthetic potentials of microbiota from diverse continents and phyla while also elucidating the biosynthetic profiles of gut archaea. Notably, our findings identify *Paenibacillus* as a dominant genus within the human gut microbiota, characterized by its extensive biosynthetic capacity. This study presents the first global atlas of BGCs within the human gut microbiome, offering valuable insights into gut-derived secondary metabolic pathways and their intricate interactions with host physiology. These results lay the groundwork for future investigations into the microbiota’s role in health and disease, underscoring the importance of understanding microbiota-derived metabolites in microbiology and gastroenterology.

**IMPORTANCE:**

This study provides a comprehensive analysis of biosynthetic gene clusters in the human gut microbiome, revealing a vast diversity of natural products with potential therapeutic applications. We identified *Paenibacillus* as a key genus with exceptional biosynthetic capabilities, including the production of leinamycin, a potent anticancer compound previously thought to be exclusive to *Streptomyces*. The findings highlight the gut microbiome as a rich, untapped resource for novel drug discovery, particularly in cancer therapy and immune modulation.

## INTRODUCTION

Microorganisms are integral to human life and production, particularly at the metabolic compound level (1). These organisms utilize specialized biosynthetic pathways to produce a wide range of compounds. Although secondary metabolites are not essential for microbial growth and reproduction, they often confer bioactive effects on host organisms, playing crucial roles in health and disease (2). These metabolites have become a cornerstone of the human pharmaceutical industry, contributing to the development of antibiotics, anticancer agents, and insecticides. The human gastrointestinal tract harbors a rich and diverse microbial community, with Firmicutes and Bacteroides being the predominant bacterial phyla. These gut microbes produce a variety of secondary metabolites, including short-chain fatty acids, bile acids, and neurotransmitters such as dopamine (3–5). These metabolites are essential for maintaining intestinal homeostasis, regulating energy metabolism, modulating immune function, and influencing aging processes.

In bacteria and fungi, secondary metabolic pathways are typically regulated by biosynthetic gene clusters (BGCs) (6–8), which encode core enzymes responsible for synthesizing the backbone metabolites, along with specialized enzymes that modify the scaffold, transcription factors, and transporter proteins that control the flow of metabolites or essential precursors (9). However, the mining of BGCs for the discovery of novel natural secondary metabolites has been hindered by the vast diversity of microorganisms, many of which cannot be cultured (10). Over the past decade, the advent of high-throughput sequencing technologies and advanced bioinformatics tools has facilitated the direct exploration of BGCs from (meta)genomes, significantly accelerating the discovery of new BGCs from previously unculturable organisms, including extremophiles (11).

Recent studies have identified numerous structural classes of BGCs, including non-ribosomal peptide synthetases (NRPS), polyketide synthases (PKS), terpenoids, and bacteriocins (12–14). Among these, NRPS and PKS are particularly attractive targets for natural product discovery due to their ability to synthesize a wide range of antibiotics and immunosuppressive agents with significant pharmacological potential. The condensation (C) domain within NRPS clusters catalyzes the formation of amide bonds, a critical step in peptide elongation, while the ketosynthase (KS) domain in PKS clusters facilitates the condensation reaction of polyketides (15). These domains are highly conserved within their respective BGC classes, making them ideal targets for genomic analyses aimed at identifying NRPS/PKS-mediated natural product pathways. Furthermore, the concept of gene cluster families (GCFs) has emerged as an effective approach for large-scale BGC analysis. This methodology involves the comparison of BGCs using pairwise distance metrics, followed by the creation of BGC families through the application of appropriate similarity thresholds (9). GCF networks have been successfully employed for global analyses of bacterial biosynthetic potential, providing valuable insights into microbial natural product diversity.

Exploring the biosynthetic potential of the human gut microbiota is crucial for understanding the complex symbiotic interactions between gut microorganisms and their human host. However, this requires comprehensive research on a global scale. In the present study, we integrate 4,744 human gut microbial genomes from the Unified Human Gastrointestinal Genome (UHGG) database to construct an extensive functional data set of BGCs. Our analysis compares the differential biosynthetic potentials of gut microbiota across diverse continents and phyla, highlighting *Paenibacillus* as a dominant genus within the human gut microbiome, characterized by its significant biosynthetic capacity, including the potential for leinamycin (LNM) synthesis.

## MATERIALS AND METHODS

### Data collection and quality assessment

In this study, a total of 4,744 human gut microbiome genomes were retrieved from the UHGG database (http://ftp.ebi.ac.uk/pub/databases/metagenomics/mgnify_genomes/human-gut/), which serves as a comprehensive resource for metagenomic data. Additionally, 785 *Paenibacillus* genomes were downloaded from the NCBI RefSeq database (https://ftp.ncbi.nlm.nih.gov/genomes/refseq/), representing a diverse collection of *Paenibacillus* strains.

To ensure the high quality of the assembled genomes, the completeness and contamination of each species-level genome bin (SGB) were evaluated using CheckM version 1.1.2 (16), a widely used tool for assessing the quality of metagenomic bins. This tool calculates the completeness of a genome as the fraction of marker genes present and contamination as the fraction of marker genes present in multiple genomic bins, which helps to ensure the integrity of each genome bin. Based on these assessments, the SGBs were classified into high-quality (completeness > 90%, contamination < 5%) or medium-quality (completeness > 50%, contamination < 5%) categories. Furthermore, an estimated quality score (QS) was calculated for each genome bin using the formula QS = completeness – 5 × contamination (17). This scoring system ensures that only high-quality genome bins, with a QS value greater than 50, were included in the analysis. All genomes meeting the high- or medium-quality criteria were retained for downstream analysis, ensuring reliable results for subsequent functional and comparative genomic investigations.

### Taxonomic assignment and phylogenetics

Taxonomic classification of each genome was performed using Genome Taxonomy Database (GTDB)-Tk version 1.4.1, which facilitates accurate taxonomic assignments based on the GTDB r95 release (18, 19). This tool relies on a set of highly conserved single-copy marker genes to assign genomes to specific taxonomic groups. For each genome, we identified the relevant single-copy marker proteins, with 122 markers for archaea and 120 markers for bacteria. These marker genes were selected for their high fidelity and broad conservation across diverse microbial taxa, providing robust taxonomic classification. The number of marker genes present in each SGB was then counted, and taxonomic assignments were made based on the presence and completeness of these markers, ensuring accurate species-level identification and classification.

To reconstruct the phylogenetic relationships among the genomes, we applied FastTree v2.1.11, a tool that uses approximate likelihood-based methods to construct phylogenetic trees from genomic data. The phylogenetic analysis was performed using the Whelan and Goldman model (20), which is well-suited for protein sequence alignments and known for its robustness in microbial phylogeny studies. FastTree’s algorithm generates a phylogenetic tree by inferring evolutionary relationships based on the alignment of marker proteins across the genomes, providing a comprehensive overview of the evolutionary landscape of the human gut microbiota. The resulting phylogenetic tree was used to investigate the relatedness between different microbial genomes and to explore the phylogenetic diversity within the human gut microbiome.

### Identification of BGCs in human gut microbial genomes

BGCs within each SGB were predicted using antiSMASH v6.0 (21), a state-of-the-art tool for identifying BGCs in microbial genomes. The prediction process was performed with a minimum overlap length of 1,000 base pairs, ensuring the identification of sufficiently large and potentially functional clusters. To enhance the accuracy of the predictions, two algorithms, ClusterFinder and KnownClusterBlast, were employed to compare the predicted BGCs against the Minimum Information about a Biosynthetic Gene Cluster database (22), which houses well-characterized biosynthetic pathways. Based on this comparison, the natural products potentially encoded by the identified BGCs were inferred, providing insights into the biosynthetic capabilities of the human gut microbiota.

To visualize the distribution and abundance of BGCs, heatmaps were generated to represent the number of genomes and BGCs across different SGBs. The heatmaps were constructed using the ggplot2 and reshape2 packages in R, which offer powerful tools for data visualization and statistical analysis. Additionally, the predicted BGCs from each genome were mapped onto a phylogenetic tree using iTOL v6 (23), enabling an intuitive visualization of the evolutionary relationships and the distribution of BGCs across the human gut microbiome.

To further explore the genomic diversity and organization of BGCs, we analyzed the frequency of gene numbers within each BGC across the 4,744 SGBs. This analysis was performed using R, which allowed for the extraction and comparison of BGC lengths in relation to the overall genome sizes. To investigate the potential effect of genome quality on the correlation between genome size and BGC number, we categorized the genomes into high-quality and medium-quality groups based on completeness and contamination thresholds. We then performed a correlation analysis using the linear regression method separately for each quality group and also conducted a comparative analysis using only complete genomes (isolated and cultured genomes).

For archaea, the obtained GenBank files were input into BiG-SCAPE, a tool designed to classify BGCs based on sequence similarity (24). The sequence similarity network was constructed using a default similarity score cutoff of 0.3 (threshold = 30%), which effectively clustered BGCs according to their gene cluster similarity. This approach facilitated the identification of potential functional relationships between different BGCs across archaeal genomes. The resulting enzyme superfamilies were then visualized using iTOL v6, providing a comprehensive and intuitive representation of the diversity of enzymatic functions encoded by the identified BGCs.

### Investigation of bacterial genera with abundant amounts of BGCs

To compare the average number of BGCs across different phyla, we accounted for genome length and quality to minimize potential biases. We selected 3,329 high-quality genomes, following established quality control criteria. To normalize BGC counts, we calculated the BGC density as follows: BGC density = BGC counts/genome length.

Additionally, to further validate the robustness of our findings, we performed a separate analysis using 1,211 complete genomes within the high-quality draft. This step aimed to assess whether the observed differences in biosynthetic potential among phyla were consistent when using only complete genomes.

The comparative analysis of biosynthetic potential between Actinobacteriota and other phyla within the human gut microbiota was performed using Fisher’s exact test. This statistical test was chosen to assess the significance of differences in the distribution of BGCs across various taxonomic groups, enabling the identification of phyla with notably higher or lower biosynthetic potential.

To identify dominant genera with high biosynthetic capabilities, genomes containing more than 15 BGCs were selected for further analysis. This threshold revealed that *Streptomyces* and *Paenibacillus* genomes exhibited the highest numbers of BGCs, suggesting these genera as key contributors to the biosynthetic diversity within the gut microbiome. To gain a deeper understanding of the biosynthetic potential of *Paenibacillus*, we downloaded 785 high-quality genomes of *Paenibacillus* strains from the NCBI database, ensuring that only complete and well-annotated genomes were included in the analysis.

These genomes were subsequently analyzed using antiSMASH v6.0 (20) to predict the BGCs present in the *Paenibacillus* genomes. In total, 12,517 BGCs were identified across these genomes. The identification of such a large number of BGCs underscores the remarkable biosynthetic capacity of *Paenibacillus* within the human gut microbiota.

For data visualization, the ggplot2 and RColorBrewer packages in R were used to generate a stacked bar chart depicting the distribution of BGCs across different classes within the *Paenibacillus* genomes. This visualization allowed for a clear comparison of the relative abundance of various BGC types within each genome, providing insights into the diversity of natural products potentially encoded by these microbial communities.

### NRPS and PKS product prediction in *Paenibacillus*

To identify potential natural product biosynthesis pathways encoded by NRPS and PKS gene clusters, we utilized the NaPDoS2 pipeline, a robust tool for the prediction and comparison of domain sequences associated with natural products. This pipeline allows for the identification of NRPS and PKS clusters by comparing their condensation (C) and ketosynthase (KS) domain sequences against domain databases of previously characterized natural products (25). The C and KS domains, which are integral to the biosynthesis of non-ribosomal peptides and polyketides, respectively, were extracted from the identified gene clusters and aligned with the NaPDoS2 database to infer the potential natural products they could synthesize.

The 785 *Paenibacillus* genomes obtained from NCBI were systematically compared to the NaPDoS2 database using the Diamond alignment tool, a fast and sensitive sequence aligner that facilitates the accurate comparison of large genomic data sets. This approach enabled the identification of NRPS and PKS gene clusters within the *Paenibacillus* genomes, linking them to specific natural product pathways. To assess the significance of the distribution of these natural products across different genera, we performed intergenus enrichment analysis on the predicted natural products. Fisher’s exact test was applied to determine whether certain natural products were significantly enriched in *Paenibacillus* compared to other genera within the human gut microbiota, providing insights into the unique biosynthetic capabilities of *Paenibacillus*.

This method offers a comprehensive view of the potential natural products produced by *Paenibacillus*, further elucidating its role in the human gut microbiome as a source of bioactive compounds.

## RESULTS

### A systematic approach identifies 8,136 biosynthetic gene clusters in UHGG database

To gain a comprehensive understanding of the biosynthetic potentials of the human gut microbiota, we recovered a total of 4,744 SGBs from the UHGG database, including 1,312 isolated and cultured genomes, 3,171 uncultured genomes, and 261 genomes with unspecified cultivation status. Of these, 3,329 SGBs were classified as high-quality drafts (>90% completeness, <5% contamination) based on recently established quality criteria (26), while the remaining genomes were deemed medium-quality (>50% completeness, <5% contamination).

**Fig 1:**
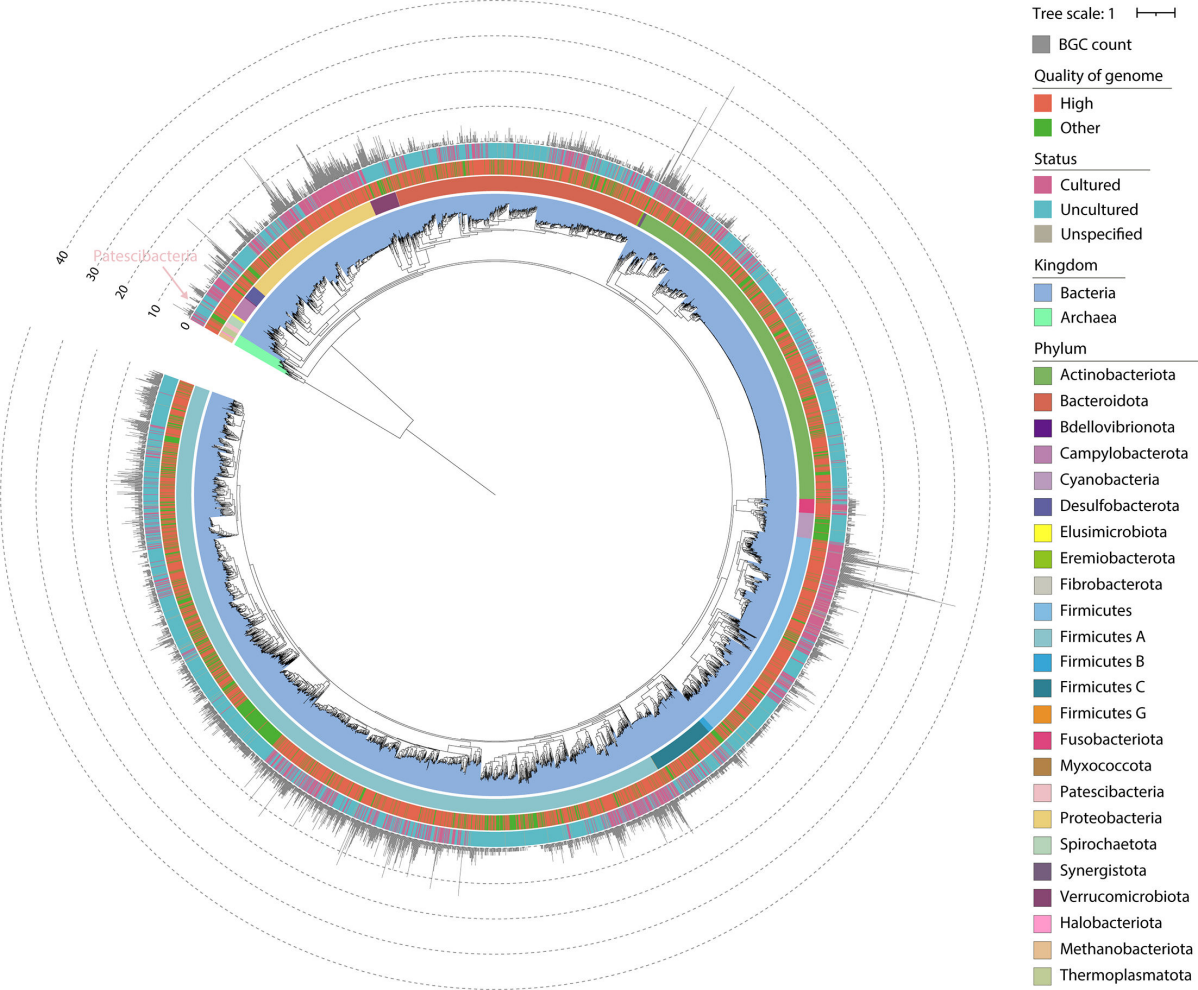
Maximum likelihood phylogenetic tree of 4,744 SGBs from the UHGG database. The phylogenetic tree was constructed using maximum likelihood methodology to depict the evolutionary relationships among 4,744 SGBs in the UHGG database. The annotation rings, moving from the inner to the outer layer, represent: (1) taxonomic classification at the kingdom level (archaea and bacteria), (2) taxonomic classification at the phylum level, (3) genome quality, (4) genome assembly status, and (5) the number of BGCs identified within each genome. Notably, BGCs were identified across all displayed phyla except for Patescibacteria, highlighting the limited biosynthetic potential of this phylum, likely attributed to its small genome size.

Metagenomic phylogenetic analysis of all genomes revealed a diverse microbial community, comprising 21 bacterial phyla and three archaeal phyla. A total of 8,136 BGCs were identified using antiSMASH, with 3,314 genomes harboring more than one BGC (Fig. 1). This extensive data set provides a robust foundation for exploring the biosynthetic diversity within the human gut microbiota and offers valuable insights into the potential production of natural bioactive compounds by these microbial communities.

The identified BGCs within the genomes ranged from 1 to 59 coding sequence regions per genome. Ninety-one percent of the genomes contained more than one gene per BGC, and 77.1% harbored at least four genes per BGC (Fig. S1A), suggesting that most predicted BGCs consist of multiple functional units. We further analyzed the relationship between genome size and BGC number by coloring each genome according to its quality (high-quality or medium-quality). The analysis revealed a noticeable trend indicating that high-quality genomes tend to show a stronger correlation between genome size and BGC number, suggesting that genome quality may influence this relationship (Fig. S1B). We also performed a separate analysis including only complete genomes to control for potential biases introduced by incomplete genome assemblies. Interestingly, even when considering only complete genomes, the correlation between genome size and BGC number remained statistically significant (*R*²= 0.26, Fig. S1C). Consistent with this, the isolated and cultured SGBs, which were classified as complete genomes, exhibited a significantly higher mean number of BGCs (2.8) compared to uncultured genomes (1.4) (Fig. S1D).

Among the 8,136 BGCs detected, the most prevalent types were ranthipeptides (1,622 BGCs, 19.9%) and cyclic-lactone autoinducers (1,269 BGCs, 15.6%), followed by RiPP recognition element (RRE)-containing BGCs (1,200 BGCs, 14.7%), NRPS BGCs (1,046 BGCs, 12.8%), and aryl polyene BGCs (489 BGCs, 6.0%) (Fig. S1E). These findings highlight the diverse array of natural product pathways encoded in the human gut microbiota. Among these, the most prominent GCF types were ribosomally synthesized and post-translationally modified peptides（RiPPs）, NRPS, and PKS, with RiPPs being the most abundant, accounting for 61.38% (Fig. S2A). Further analysis of the taxonomic distribution at the phylum level revealed that the predominant phylum within NRPS was Firmicutes A, while Actinobacteriota dominated polyketide synthase I (PKSI), and Firmicutes A was also the most prevalent phylum within RiPPs (Fig. S2B).

### Comparison of human gut microbial biosynthetic potential across continents

**Fig 2:**
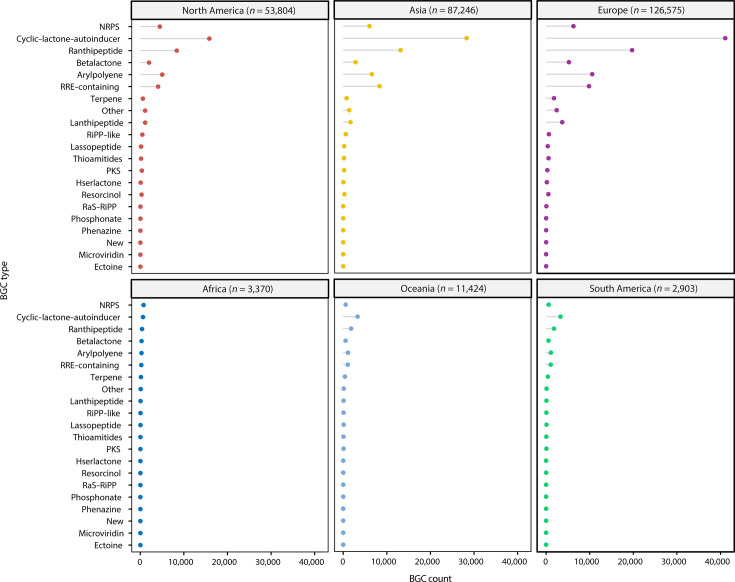
Statistics of the BGCs predicted in the MAGs from six continents. The top 21 BGC types ranked by genomic frequency are shown, and unclassified BGCs are labeled as new. *n*, number of MAGs from each continent.

Utilizing metagenome-assembled genomes (MAGs) corresponding to the 4,744 SGBs, we investigated the biosynthetic potential of the human gut microbiota across various continents. Following the prediction of BGCs in the global human gut microbiomes, we quantified the number of BGCs within each cluster type across different continents (Fig. 2). It shows the geographic distribution of BGCs across human gut microbial genomes. The classification of genomes by geographic location was based on metadata provided by the UHGG database, which specified the country of isolation. The distribution pattern observed in Fig. 2 reflects the geographic categorization from the UHGG data. In regions with a relatively high abundance of MAGs (*n* > 10,000), including Europe, Asia, North America, and Oceania, we observed a broadly consistent distribution of BGC types. As seen in our SGB-level analysis (Fig. S1D), cyclic-lactone autoinducers and ranthipeptides, both linked to peptide synthesis, were the most prevalent BGCs across all continents. A notable exception, however, was the RRE-containing BGCs, which exhibited a significantly higher relative abundance in Asia compared to other regions. This was especially pronounced in the Northern Hemisphere, where MAG counts exceeded 50,000 (Fig. 2).

### Exploration of archaeal biosynthetic potentials in human gut microbiota

Among the 4,744 SGBs from the UHGG database, 28 were classified into archaeal phyla, including Thermoplasmatota, Methanobacteriota, and Halobacteriota. Given that the secondary metabolic potential of archaeal phyla remains largely unexplored, we aimed to systematically evaluate the biosynthetic capabilities of human gut archaea by conducting a comprehensive analysis of these recovered genomes.

**Fig 3:**
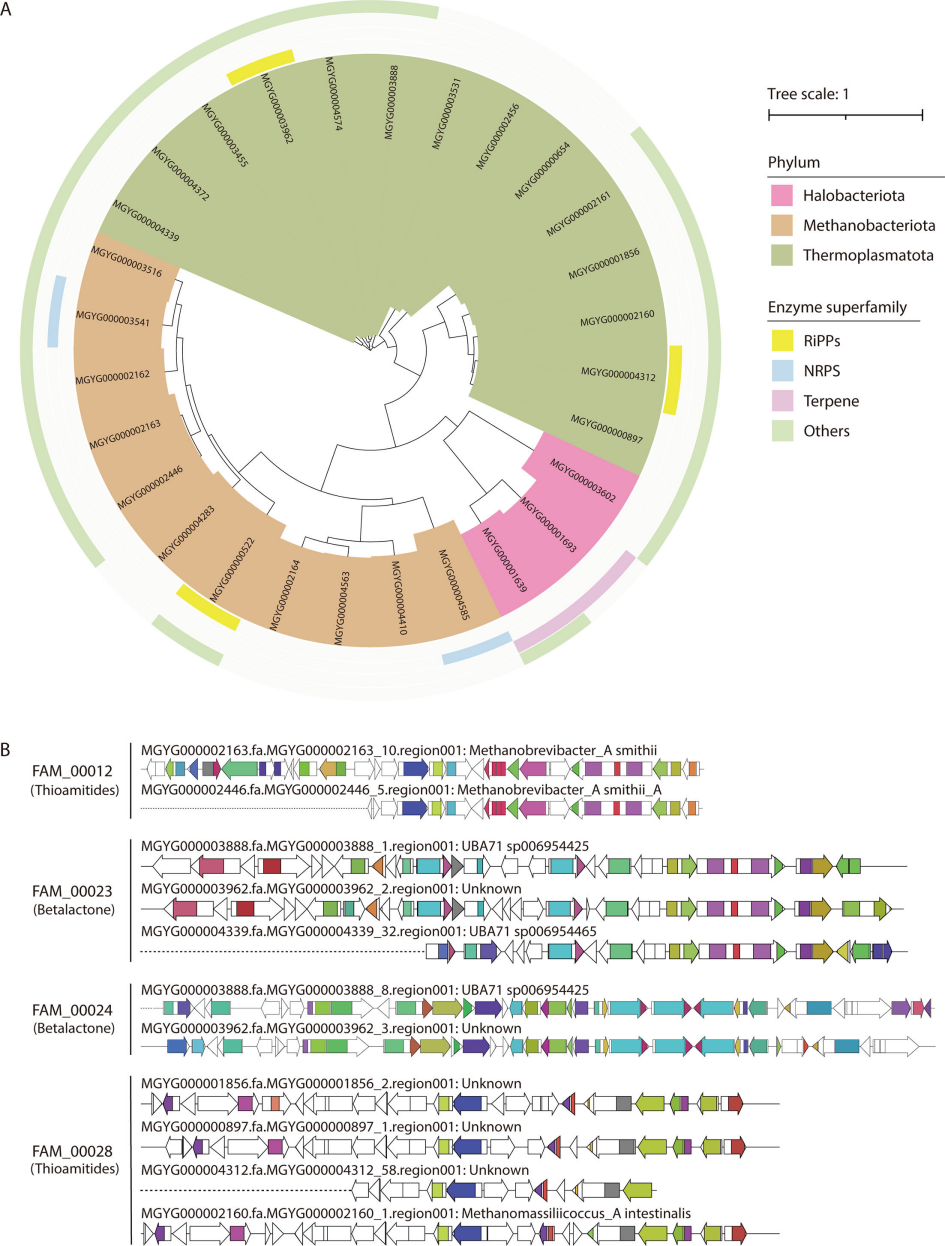
Biosynthetic capacity prediction of archaea. (**A**) BiG-SCAPE clustering distribution of 28 archaeal SGBs from UHGG. The enzyme superfamilies of BGCs predicted in these archaeal SGBs are labeled. (**B**) Four GCFs consisting of multiple BGC members in the archaeal SGBs. All of these GCFs pertain to the Others enzyme superfamily, with potentials to synthesize thioamide or betalactone products (labeled correspondingly in the brackets).

A total of 34 BGCs were predicted across the 28 archaeal SGBs, belonging to four enzyme superfamilies: RiPPs, NRPS, Terpenes, and Others (Fig. 3A). Based on the gene cluster structure, 27 GCFs were identified for these archaeal BGCs. Notably, four GCFs—FAM_00012, FAM_00023, FAM_00024, and FAM_00028—within the “Others” enzyme superfamily exhibited clustering of multiple BGC members (Fig. S3). Further structural analysis of the BGCs within these four GCFs revealed their potential to synthesize thioamides (FAM_00012 and FAM_00028) and betalactones (FAM_00023 and FAM_00024), despite significant structural differences between the BGCs predicted to produce the same class of compounds (Fig. 3B).

### Comparison of human gut microbial biosynthetic potential across phyla

**Fig 4:**
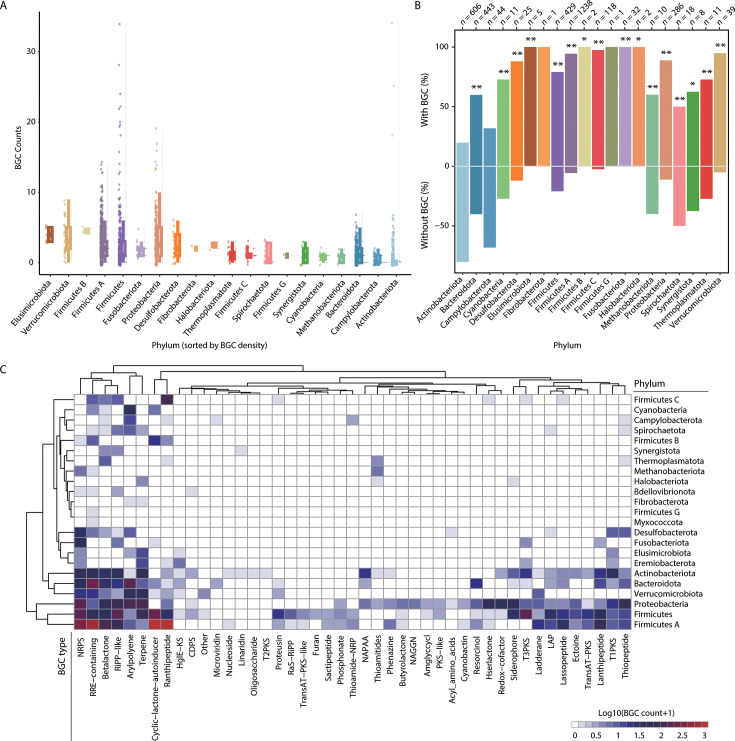
Comparison of biosynthetic potentials across the phyla identified in UHGG genomes. (**A**) Distribution of predicted BGC counts per SGB across the identified phyla. The top 20 phyla are ranked by the mean BGC count per genome, from highest to lowest. (**B**) Percentage of SGBs containing or lacking predicted BGCs for each phylum. Fisher’s exact test was used to compare the biosynthetic potentials of Actinobacteriota with the other 19 phyla, with statistical significance indicated by asterisks (*, fdr < 0.05; **, fdr < 0.01). *n* refers to the number of SGBs within each phylum, which is also applicable to the phyla analyzed in panel **A**. (**C**) Heatmap showing the distribution of BGC counts for different biosynthetic cluster types across the various phyla.

Among the 24 bacterial and archaeal phyla identified in the UHGG SGBs, 23 phyla were predicted to harbor biosynthetic potential, with the exception of the bacterial phylum Patescibacteria (Fig. 1). To further investigate the biosynthetic potential across these phyla, we calculated the BGC density while controlling for genome length. The analysis was confined to 3,329 high-quality genomes to minimize the influence of genome quality on the results. Remarkably, Elusimicrobiota exhibited the highest average number of BGCs per genome (>4), suggesting its prominent biosynthetic capacity (Fig. 4A). Overall, a substantial proportion of SGBs across most phyla contained predicted BGCs (Fig. 4B). Among these, Actinobacteriota displayed the lowest proportion of SGBs harboring BGCs (19.8%). Statistical analysis using Fisher’s exact test further confirmed that the biosynthetic potential of the remaining 16 phyla was significantly greater than that of Actinobacteriota in the human gut microbiome (Fig. 4B). Furthermore, we performed a targeted analysis of 1,211 complete genomes within the set of 3,329 high-quality genomes to validate the consistency of the observed patterns (Fig. S4). Collectively, while Actinobacteriota is known for its synthesis of various secondary metabolites, its contribution to the biosynthetic landscape of the human gut microbiome appears to be relatively limited. We subsequently analyzed the enrichment of BGC types across all phyla (Fig. 4C) and found that the dominant phyla in the human gut microbiota—Firmicutes, Firmicutes A, Bacteroidota, Proteobacteria, Actinobacteriota, and Verrucomicrobiota—demonstrated notable biosynthetic potentials. These phyla exhibited both the highest diversity of enriched BGC types and significantly higher overall enrichment degrees compared to other phyla. Specifically, NRPS clusters were markedly enriched in Firmicutes, Firmicutes A, and Proteobacteria. Peptide-related BGC types, such as RRE-containing, cyclic-lactone autoinducers, ranthipeptides, lassopeptides, and lanthipeptides, were most highly enriched in Firmicutes A, which showed the strongest enrichment for these types. Terpene BGCs were preferentially enriched in Firmicutes, Proteobacteria, Verrucomicrobiota, and Actinobacteriota. Furthermore, among the archaeal phyla, thioamide production was collectively enriched in Thermoplasmatota, Halobacteriota, and particularly Methanobacteriota.

### Identification of *Paenibacillus* as a bacterial genus with dominant biosynthetic potential

**Fig 5:**
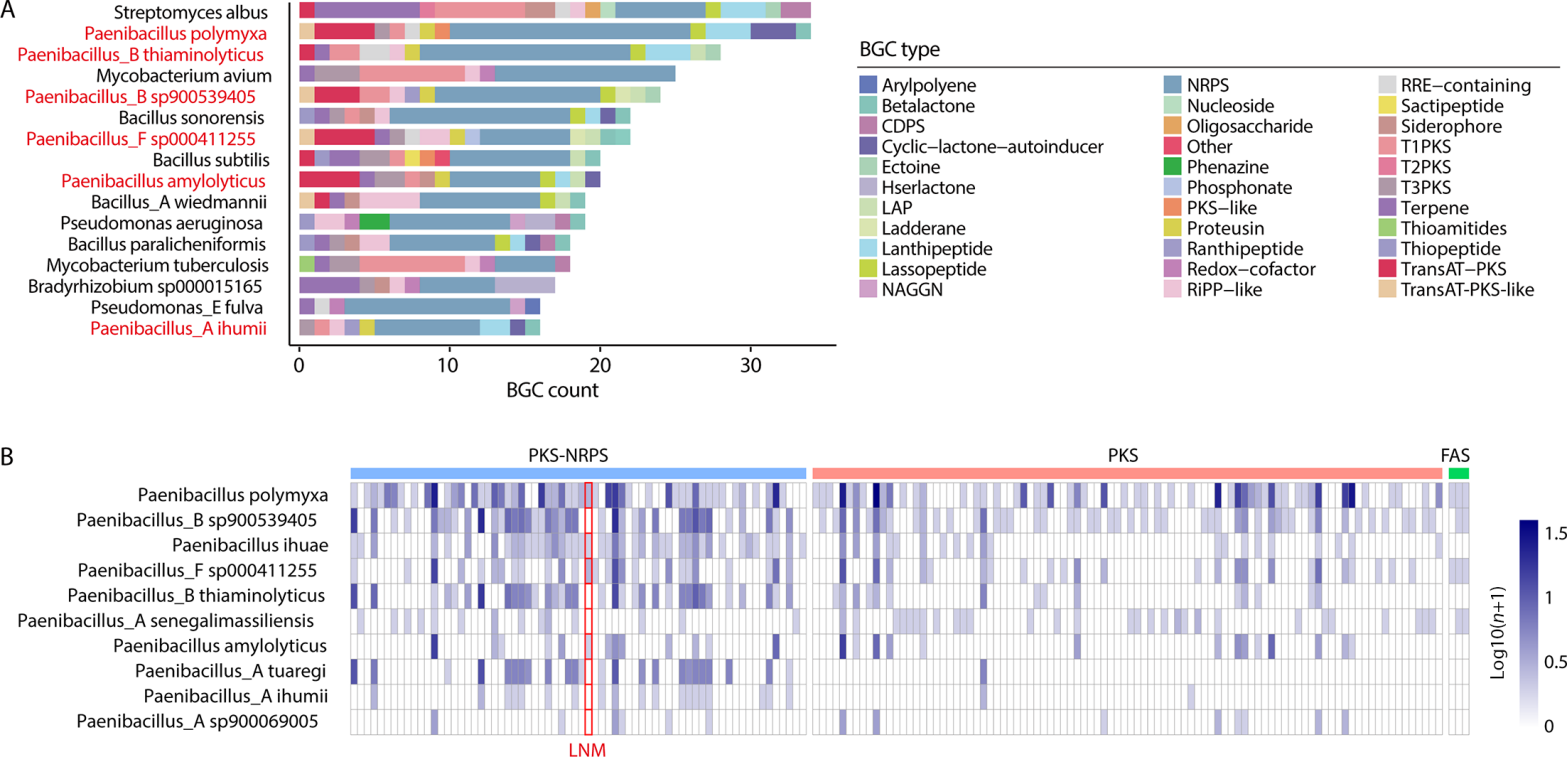
Identification of *Paenibacillus* as a human gut bacterial genus possessing significant biosynthetic potentials. (**A**) Statistics of BGC counts (>15) and types predicted in the UHGG SGBs with a rank of high-low BGC count. The strains of SGBs are annotated, with *Paenibacillus* strains labeled (red). (**B**) Abundances of biosynthetic potentials for the predicted secondary metabolites synthesized by the PKS and NRPS BGCs in *Paenibacillus* SGBs. Ten *Paenibacillus* SGBs from the UHGG are predicted to synthesize PKS- and NRPS-related secondary metabolites, which overall involve the metabolic pathways of FAS, PKS, and PKS-NRPS. The strains are ranked by the total number of predicted secondary metabolite types within them. Data related to the natural product LNM are labeled (red). *n*, number of KS or C domains matched to reference sequences for each natural product.

Building on the identification of SGBs with a remarkable number of predicted BGCs (Fig. 4A), we next focused our analysis on strains exhibiting significant biosynthetic potential. Among the 4,744 SGBs, 16 genomes contained more than 15 predicted BGCs, which were confined to three bacterial phyla: Actinobacteriota (specifically *Streptomyces* and *Mycobacterium*), Firmicutes (including *Paenibacillus* and *Bacillus*), and Proteobacteria (including *Pseudomonas* and *Bradyrhizobium*) (Fig. 5A). Notably, the sole *Streptomyces albus* SGB in the UHGG data set exhibited the highest number of BGCs (34) and the greatest diversity of BGC types (14), in line with the well-documented exceptional biosynthetic capabilities of this genus. Interestingly, an SGB of *Paenibacillus polymyxa* also harbored 34 predicted BGCs, and up to half of the top 10 SGBs ranked by BGC count were attributed to *Paenibacillus*, strongly suggesting that *Paenibacillus* is a genus with dominant biosynthetic capacities within the human gut microbiota.

To further explore the secondary metabolic potentials of *Paenibacillus*, we collected 785 genomes of this genus from the NCBI database, resulting in the prediction of a total of 12,517 BGCs (16 BGCs per genome) (Table S1). Among the predicted BGC types, NRPS were the most abundant, accounting for 37.5% (4,702), followed by cyclic-lactone autoinducers (8.7%, 1,086), trans-acyltransferase PKS (8.5%, 1,063), and terpenes (5.6%, 703). Notably, our analyses also identified several BGC types previously unreported in *Paenibacillus*, such as lassopeptides (3.9%, 483) and phosphonates (1.3%, 162), thereby expanding the known biosynthetic repertoire of this genus. Additionally, two strains, *P. polymyxa B_3* (97.52% completeness, 2.25% contamination) and *Paenibacillus B sp001894745* (98.76% completeness, 0.2% contamination), were found to harbor the highest numbers of BGCs, with 61 and 55 predicted clusters, respectively. These findings underscore the substantial secondary metabolic potential of *Paenibacillus* and highlight these strains as high-priority candidates for further investigation.

### Prediction of PKS and NRPS products synthesized by *Paenibacillus*

PKS and NRPS, including their hybrid PKS-NRPS complexes, are among the most structurally diverse families of natural products, known for their wide-ranging biological activities. These pathways provide a rich source of clinically significant drugs, including antibiotics and anti-tumor agents (27, 28). In this study, we investigated the biosynthetic potential of PKS and NRPS products in 18 *Paenibacillus* SGBs from the UHGG database. Of these, 10 SGBs were predicted to synthesize PKS- or NRPS-derived metabolites, encompassing a total of 165 distinct metabolite types. These were distributed across three major synthetic pathways: PKS (94, 57.0%), PKS-NRPS hybrids (68, 41.2%), and fatty acid synthase (FAS; 3, 1.8%) (Fig. 5B). Notably, the *P. polymyxa* strain exhibited the highest number of predicted PKS and NRPS metabolites (106), further reinforcing its remarkable biosynthetic potential. This finding aligns with its exceptional number of predicted BGCs, which ranked the highest among all the SGBs recovered from the UHGG data set (Fig. 5A).

**Fig 6:**
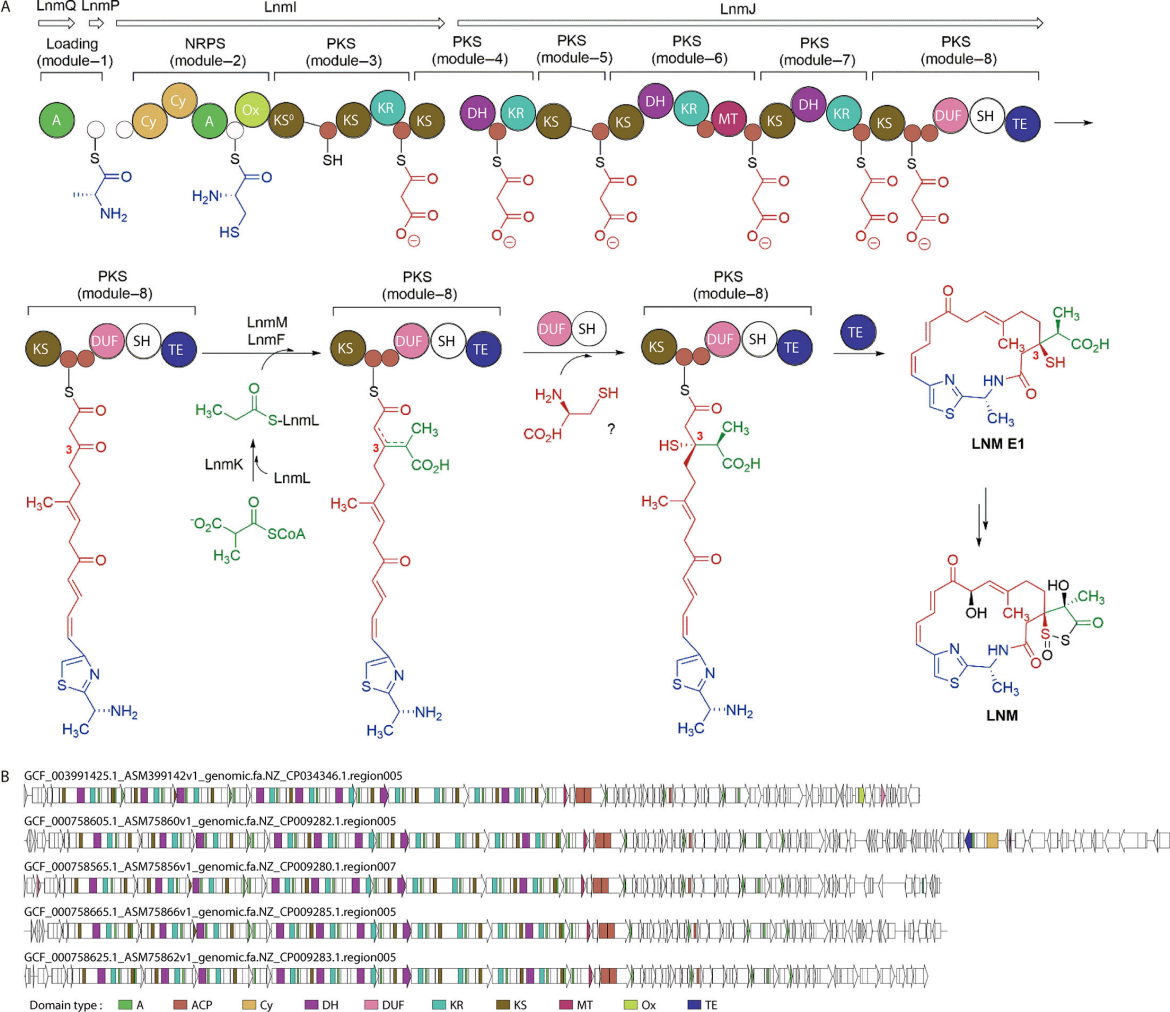
Structural analysis on the LNM BGCs predicted in *Paenibacillus*. (**A**) Mechanism of LNM biosynthesis reported in *Streptomyces atroolivaceus S-140*. The image is obtained via modification based on the original drawing in reference (29). The LNM biosynthetic machinery involves a hybrid NRPS-AT-less type I PKS (LnmQ, P, I, J), a discrete acyl transferase (AT; LnmG) that loads malonyl-coenzyme A as an extender unit onto all six PKS modules, and a set of enzymes (LnmK, L, M, F) responsible for the installation of β-alkyl branch at C3 position. After 3-thiol group incorporation, off-loading from the PKS, and tailoring enzyme modification, the natural product LNM is eventually obtained. The different biosynthetic origins of the LNM molecule are colored for NRPS (blue), PKS (red), β-alkyl branching cassette (green), and other tailoring enzymes (black). A, adenylation protein; Cy, condensation/cyclization domain; DH, dehydratase domain; DUF, domain of unknown function; KR, ketoreductase domain; KS, ketosynthase domain; KS^0^, non-elongating KS domain; MT, methyltransferase domain; Ox, oxidation domain; SH, cysteine lyase domain; TE, thioesterase domain; empty circle, peptidyl carrier protein; solid red circle, acyl carrier protein. (**B**) Genetic organization of 5 LNM BGCs predicted in *Paenibacillus*. These selected BGCs possess the highest identity scores (>50%) to the reference protein sequences of the LNM KS domain, in the group predicted with *Paenibacillus* genomes containing only one contig. The domain types identified in panel **B**, which are also included in the reported major process of LNM biosynthesis as indicated in panel **A**, are colored correspondingly in both drawings.

LNM is a macrolide antibiotic characterized by a distinctive 1,3-dioxo-1,2-dithiolane moiety, which is helically fused to an 18-membered macrocyclic lactam ring (Fig. 6A). This biomolecule holds significant clinical importance due to its anti-cancer properties and has so far only been reported in *Streptomyces* for natural biosynthesis. Interestingly, LNM was predicted as a product of the PKS-NRPS biosynthetic pathway in *Paenibacillus* SGBs from the UHGG database (Fig. 5B), with six LNM-specific BGCs identified across four distinct SGBs. Extending this analysis, we explored the 785 *Paenibacillus* genomes from the NCBI database, uncovering a total of 255 LNM-related BGCs. To further characterize these pathways, we performed clustering analysis based on protein sequence identities for all 261 LNM BGCs predicted in both the UHGG and NCBI *Paenibacillus* genomes using BiG-SCAPE. This analysis identified 59 GCFs within the PKS-NRPS enzyme superfamily. Among these, 28 GCFs contained multiple BGC members, and 11 of these GCFs were further grouped into four gene cluster clans (GCCs) (Fig. S5).

To more accurately assess the structural composition of the predicted LNM BGCs in *Paenibacillus*, we focused on those identified in NCBI genomes containing a single contig, thereby minimizing the potential interference of genomic incompleteness in BGC prediction. Notably, the top five BGCs in this subset, ranked by their high identity (>50%) to the reference protein sequences of the LNM KS domain, were all grouped within GCC_084. This group consisted of four BGCs in FAM_00148 and one in FAM_00214 (Fig. S5), suggesting that this GCC is particularly relevant for further analysis. Functional annotation of the BGCs using BiG-SCAPE revealed that the major domains involved in LNM biosynthesis, as reported in *Streptomyces atroolivaceus* S-140, were present in these five *Paenibacillus* genomes. These domains included adenylate cyclase, acyl carrier protein, condensation/cyclization, oxidation, and key enzymes such as dehydratase, methyltransferase, ketoreductase, KS, and thioesterase (Fig. 6A and B). Moreover, consistent with the structural features of Streptomyces LNM BGCs, the domains of ketoreductase, KS, and dehydratase were repeatedly identified and co-located within the five *Paenibacillus* BGCs. Collectively, these findings represent the first identification of LNM biosynthetic potential beyond *Streptomyces*, shedding light on the abundant secondary metabolic capabilities of *Paenibacillus*.

## DISCUSSION

This study leverages the extensive genomic data from the Human Microbiome Project and other global microbiome sequencing efforts to systematically explore the biosynthetic potential embedded within the human gut microbiota. By analyzing 4,744 SGBs from the UHGG database, we have identified a remarkable diversity of BGCs, shedding light on the untapped natural product potential of the human gut microbiome. These findings significantly extend our understanding of the functional roles of gut microbes in human health, highlighting the potential of the microbiome as a source of bioactive compounds that could influence host physiology and disease resistance.

Our results align with previous studies suggesting that the human gastrointestinal tract is predominantly colonized by Firmicutes and Bacteroidetes (30), which together account for 67.9% of the recovered genomes in our study. These two phyla, which are the most abundant in the human gut, appear to play a central role in secondary metabolite biosynthesis. This is consistent with their well-established role in metabolizing complex carbohydrates and synthesizing a variety of bioactive compounds such as short-chain fatty acids and antimicrobial peptides (31). The high abundance of Firmicutes and Bacteroidetes in the gut microbiome suggests that they are key contributors to maintaining intestinal health and modulating host immune responses through their biosynthetic activity (32, 33).

Interestingly, our findings revealed some overlap between the high-abundance BGCs identified in our study and those reported in previous literature, particularly in clusters related to NRPS (34). Additionally, we identified several BGCs with high abundance that were not reported in previous studies, which may be attributed to differences in sample origin and analysis strategy. A significant portion of the BGCs identified in our study were involved in the biosynthesis of RiPPs, which include lanthipeptides, lassopeptides, and ranthipeptides (35). These classes of secondary metabolites are known for their antimicrobial and immunomodulatory activities (34), and our findings confirm their widespread presence in Firmicutes and Actinomycetes. Interestingly, we also found Proteobacteria and Verrucomicrobiota to harbor lanthipeptide BGCs, suggesting that RiPPs may be more broadly distributed in the human gut microbiota than previously appreciated. These findings extend our understanding of the metabolic diversity within the microbiome, which likely contributes to a wide range of health benefits, including antimicrobial defense and modulation of gut microbiota composition.

One of the more intriguing discoveries in this study was the identification of thioamide BGCs in Archaea, particularly in Methanobacteriota. Thioamides are rare post-translational modifications catalyzed by the YcaO-TfuA protein pair (36), and their enrichment in archaeal phyla may suggest a unique biochemical role in microbial metabolism or host-microbe interactions. This discovery provides new insights into the metabolic potential of archaea in the human gut, which have been less studied compared to bacteria and fungi but may contribute to key metabolic functions within the microbiome.

In addition to the global perspective, we conducted a comparison of biosynthetic potentials across different continents, including Europe, Asia, North America, and Oceania, based on the MAG-level BGC predictions. Our analysis revealed a broadly consistent distribution of BGC types across continents, suggesting that the core biosynthetic functions of the human gut microbiota are conserved across diverse human populations. However, we also observed regional differences in certain BGC types, such as a higher relative abundance of RRE-containing BGCs in the gut microbiota of individuals from Asia. This regional variation may be related to dietary habits, environmental factors, or genetic differences, consistent with previous research highlighting the significant variability in gut microbiome composition across geographic regions (37). These findings point to the possibility that regional microbiomes may harbor distinct biosynthetic capacities, which could have implications for the development of tailored microbiome-based therapies.

One of the most novel findings of our study was the identification of *Paenibacillus* as a dominant genus with significant biosynthetic potential within the human gut microbiota. This genus, previously less studied in the context of gut health (38), was found to harbor a wide array of NRPS- and RiPPs-related BGCs, in addition to BGCs responsible for ectoine and terpene biosynthesis. *Paenibacillus* species have been recognized for their capacity to produce various bioactive compounds, and our study suggests that they may contribute to gut health through the production of antimicrobial and immunomodulatory metabolites. More importantly, we also identified an LNM biosynthetic pathway in *Paenibacillus*, a compound previously thought to be restricted to *Streptomyces* (29, 39). LNM is a promising anti-cancer agent, known for its ability to target tumors resistant to conventional therapies (40, 41). The potential natural presence of LNM in the human gut microbiota raises intriguing questions about its role in gut immunity and overall health, suggesting that *Paenibacillus* could play a previously unrecognized role in cancer prevention or treatment.

Despite the valuable insights this study provides into the biosynthetic landscape of the human gut microbiome, several limitations must be acknowledged. While this study primarily focuses on the exploration of NRPS and PKS class secondary metabolites, RiPPs, which represent the largest proportion of the 4,744 predicted BGCs in the SGBs, have garnered significant attention due to their unique chemical structures and diverse biological activities. Future research will expand on this class of secondary metabolites. The accuracy of BGC predictions is highly contingent on the completeness and quality of genome assemblies; incomplete genomes may lead to an underestimation of the true biosynthetic potential of certain microbes. Additionally, although *in silico* predictions provide valuable insights, they require experimental validation to confirm the production of the predicted metabolites. Without isolation and characterization of these compounds, their bioactivity and potential therapeutic applications remain speculative. Furthermore, this study did not integrate BGC data with metabolomics data, a combination that could offer a more holistic understanding, particularly in the context of the high number of BGCs observed in *Paenibacillus*.

Future research should focus on improving genome assembly completeness, especially for underrepresented or less characterized taxa, to enhance the accuracy of BGC predictions. It is crucial to experimentally validate the predicted metabolites through isolation and characterization to better understand their bioactivity. Moreover, integrating BGC data with metabolomics data, especially in *Paenibacillus*, will provide a more holistic view of the microbiome’s biosynthetic capacity and its physiological implications. Longitudinal studies investigating the effects of diet, lifestyle, and disease states on the microbiome’s biosynthetic potential will be essential to determine how these metabolites contribute to human health and disease prevention. Understanding the functional roles of microbiome-derived metabolites in human health should be a key focus of future work.

While this study has its limitations, particularly in the lack of integration between genomic and metabolomic data, its findings highlight the untapped biosynthetic potential of the human gut microbiota. The discovery of microbial players like *Paenibacillus*, which produce novel metabolites such as LNM, offers significant implications for microbiome-based therapeutic strategies, especially in areas like cancer treatment and immune modulation. This research lays the groundwork for future studies that can further explore the diverse array of natural products in the gut microbiome, providing important insights into their role in human health.

## SUPPLEMENTAL MATERIAL

Fig. S1 msystems.00610-25-s0001.tif The relationship between the quality size of 4,744 genomes and the type and count of BGCs.

Fig. S2 msystems.00610-25-s0002.tif Classification and distribution of BGCs into GCFs.

Fig. S3 msystems.00610-25-s0003.tif Similarity network of 27 GCFs in Archaea.

Fig. S4 msystems.00610-25-s0004.tif Comparison of biosynthetic potential between Actinobacteriota and other phyla.

Fig. S5 msystems.00610-25-s0005.tif Clustering analysis of the protein sequence characteristics of all 261 predicted LNM BGCs from the UHGG and NCBI *Paenibacillus* genomes using BiG-SCAPE.

Legends msystems.00610-25-s0006.docx Legends for supplemental items.

Table S1 msystems.00610-25-s0007.xlsx Results of the identification of 785 *Paenibacillus* genomic biosynthetic gene clusters.

ASM does not own the copyrights to Supplemental Material that may be linked to, or accessed through, an article. The authors have granted ASM a non-exclusive, world-wide license to publish the Supplemental Material files. Please contact the corresponding author directly for reuse.
