# Supplementary figures and images for: A comprehensive analysis of human gut microbial biosynthesis gene clusters unveiling the dominant role of Paenibacillus
Source: mSystems. 2025 Jun 9;10(7):e00610-25. doi: 10.1128/msystems.00610-25 (PMC12282132; doi:10.1128/msystems.00610-25)

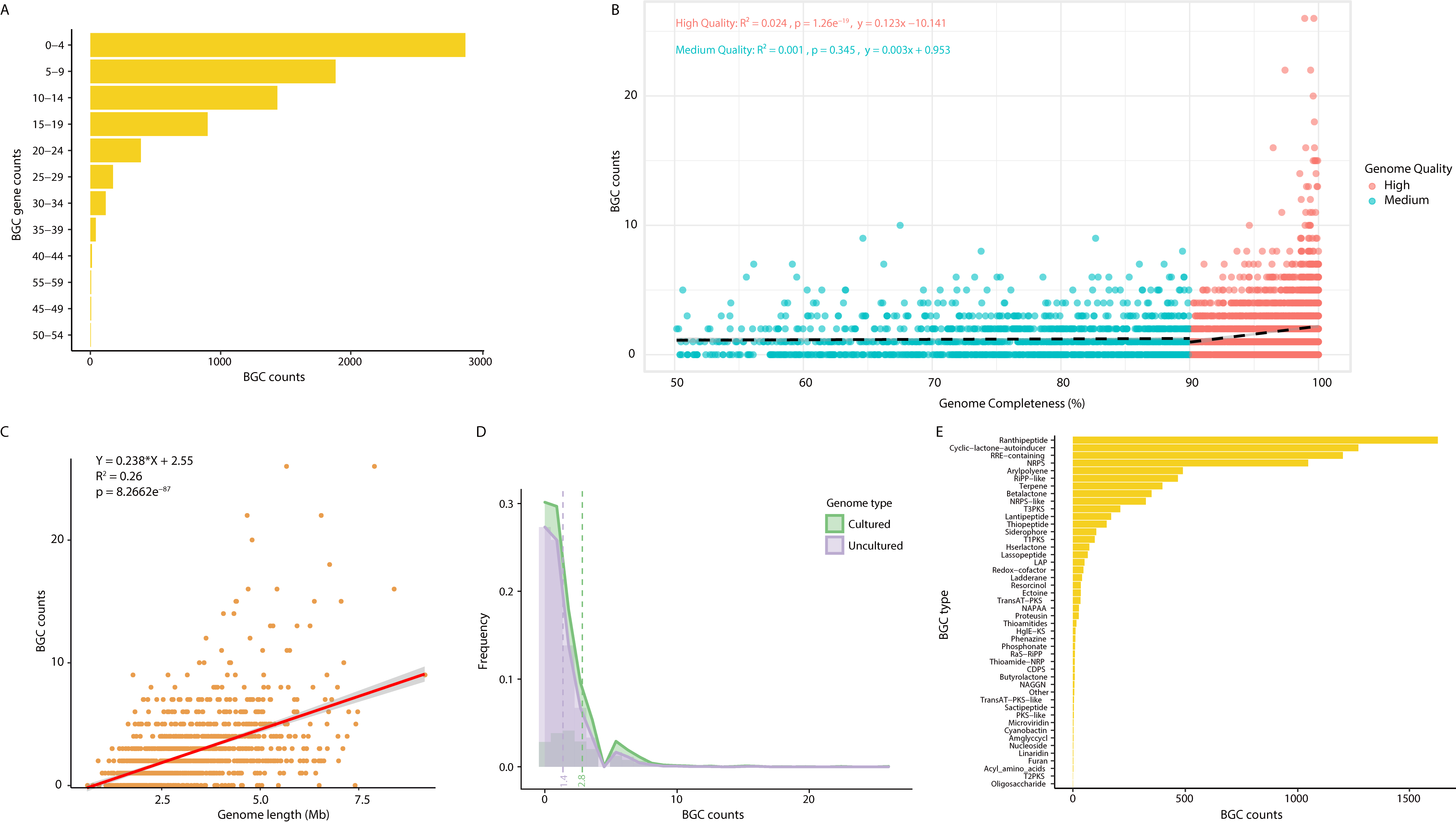

Supplement: Fig. S1 — The relationship between the quality size of 4,744 genomes and the type and count of BGCs. [file msystems.00610-25-s0001.tif]

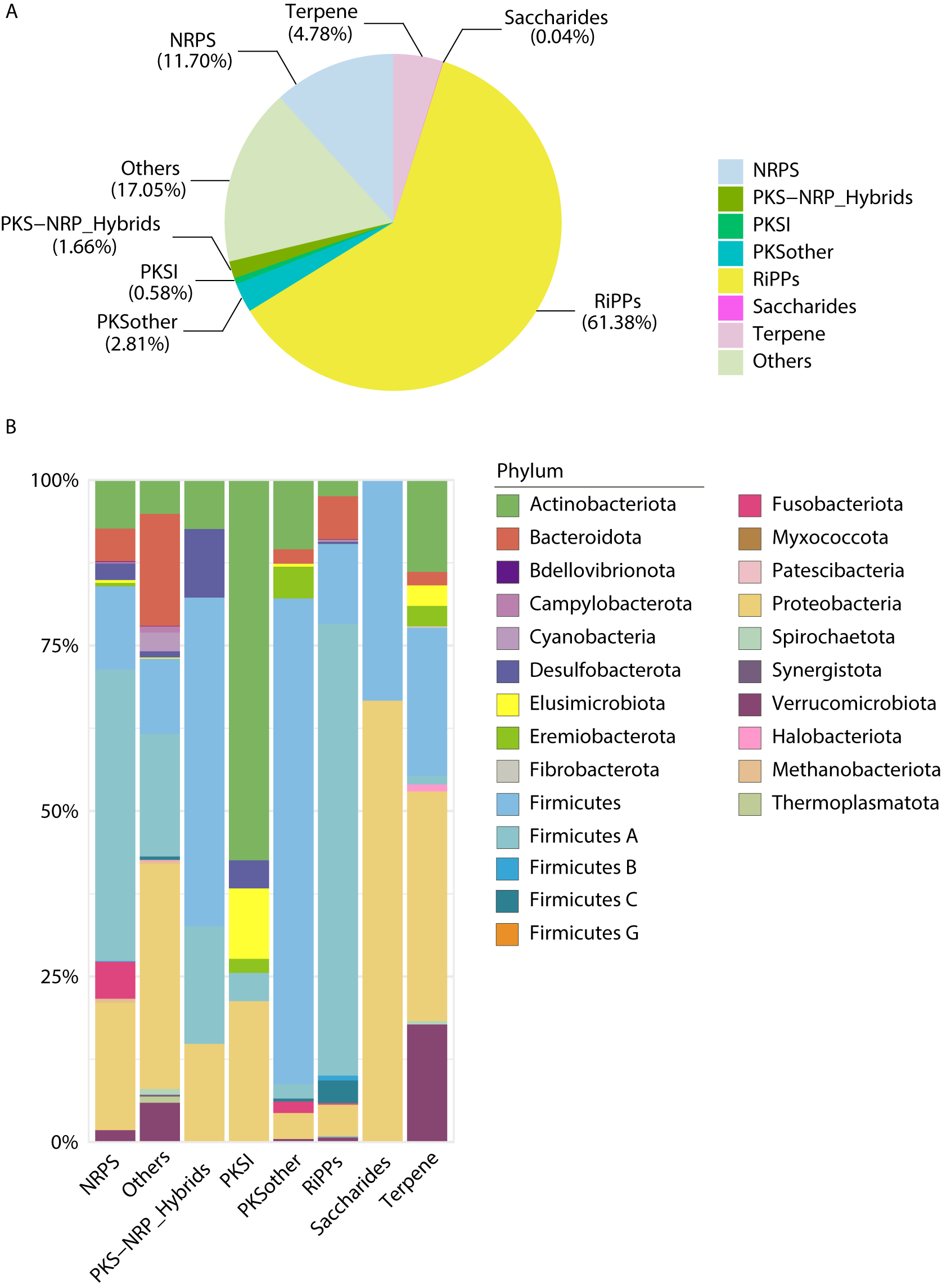

Supplement: Fig. S2 — Classification and distribution of BGCs into GCFs. [file msystems.00610-25-s0002.tif]

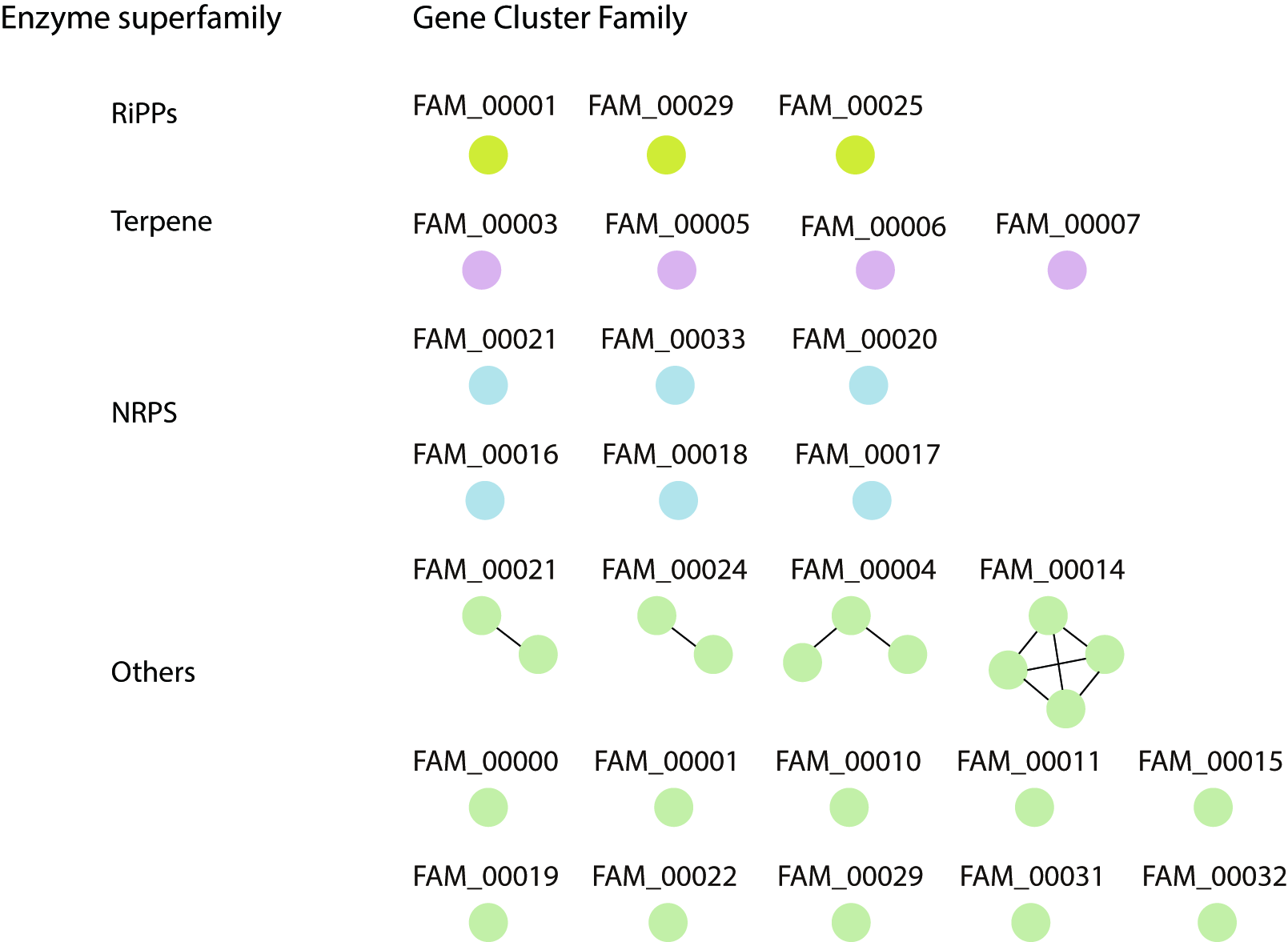

Supplement: Fig. S3 — Similarity network of 27 GCFs in Archaea. [file msystems.00610-25-s0003.tif]

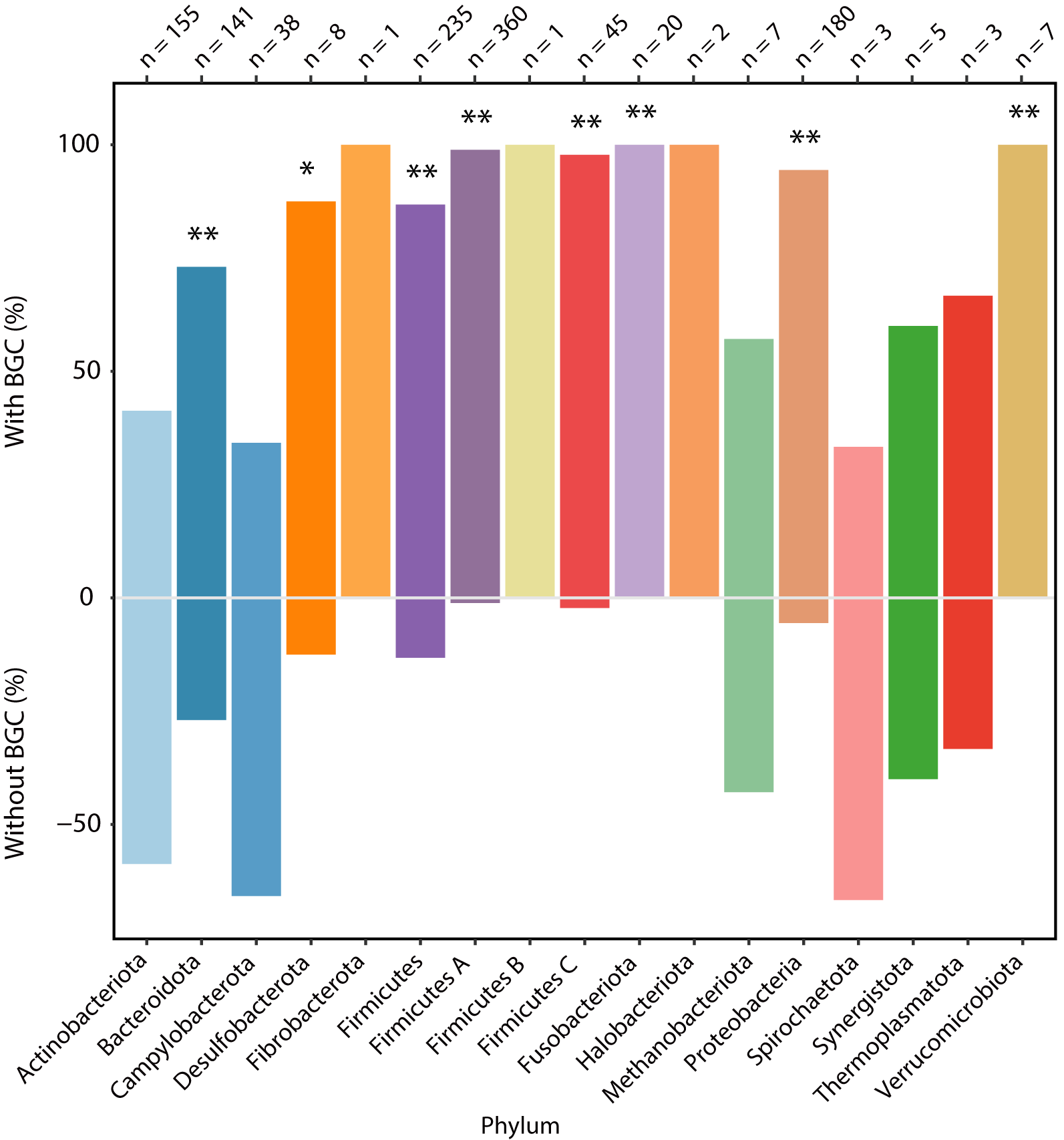

Supplement: Fig. S4 — Comparison of biosynthetic potential between Actinobacteriota and other phyla. [file msystems.00610-25-s0004.tif]

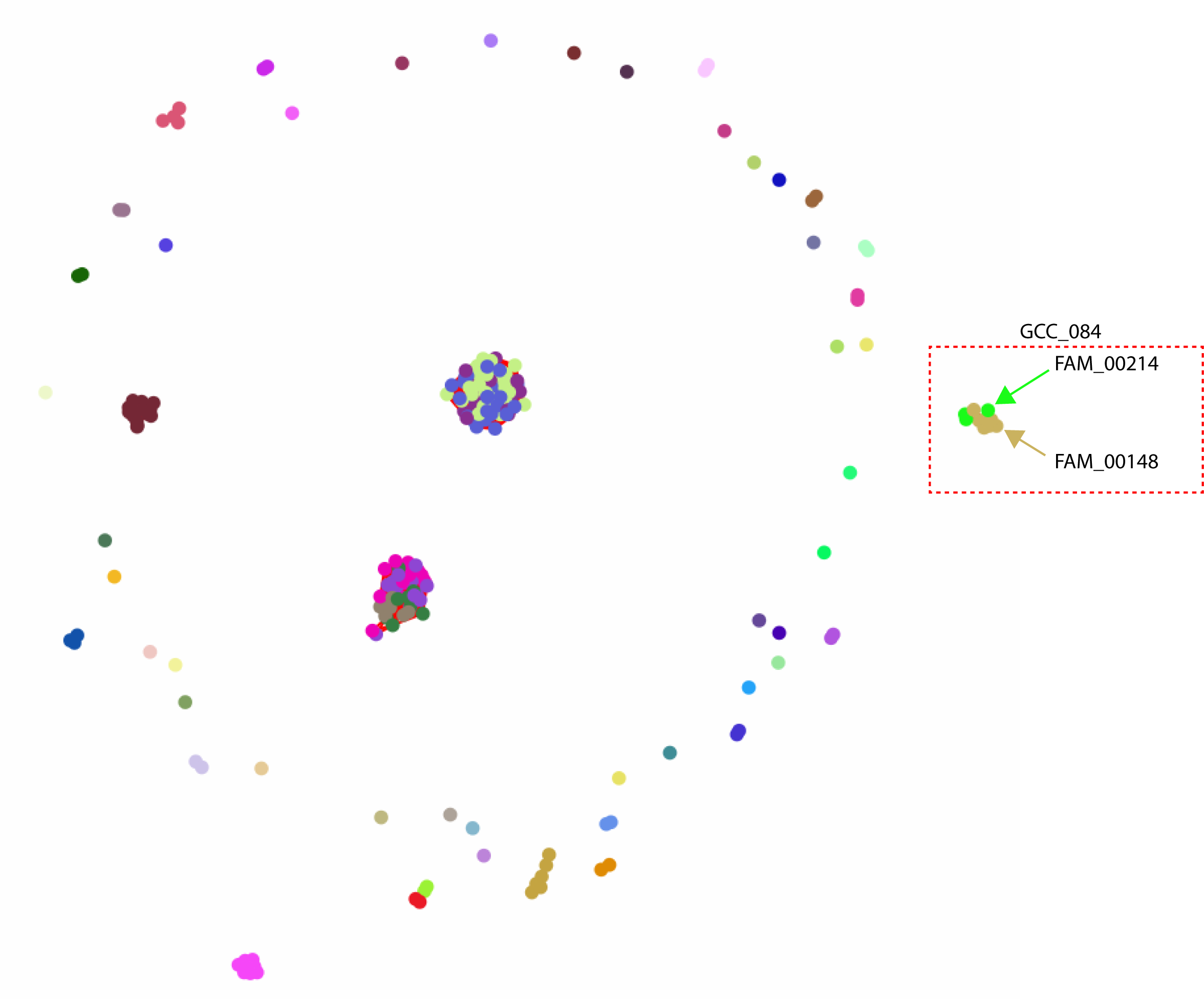

Supplement: Fig. S5 — Clustering analysis of the protein sequence characteristics of all 261 predicted LNM BGCs from the UHGG and NCBI Paenibacillus genomes using BiG-SCAPE. [file msystems.00610-25-s0005.tif]
