# Supplementary material for: A comprehensive analysis of human gut microbial biosynthesis gene clusters unveiling the dominant role of Paenibacillus
Source: mSystems. 2025 Jun 9;10(7):e00610-25. doi: 10.1128/msystems.00610-25 (PMC12282132; doi:10.1128/msystems.00610-25)
Supplement: Legends — for supplemental items. [file msystems.00610-25-s0006.docx]

**Supplementary Information**

**Table S1**. **Results of the identification of 785 Paenibacillus genomic biosynthetic gene clusters.**

**Figure S1.** **The relationship between the quality size of 4744 genomes, the type and count of BGCs.** (A). Number of genes in the CDS region encoding biosynthetic gene clusters (BGCs). (B). Relationship between genome completeness and the number of BGCs. (C). Relationship between genome size and the total number of BGCs in each genome. (D). Number of biosynthetic gene clusters in the genome of whether or not they were isolated and cultured. (E). Number of biosynthetic gene clusters per type.

**Figure S2.** **Classification and distribution of BGCs into GCFs.** (A). The distribution of the 8136 BGCs across 8 GCFs, showing the proportional representation of each GCF. (B). Phylum-level classification of the SGBs used to predict the BGCs in each GCF, illustrating the phylum origins of the SGBs that were used for BGC prediction.

**Figure S3.** **Similarity network of 27 GCFs in Archaea.**

**Figure S4.** **Comparison of biosynthetic potential between Actinobacteriota and other phyla.** Statistical comparison of biosynthetic potential between Actinobacteriota and other phyla, based on predicted BGCs from 1211 complete genomes. Fisher’s exact test was used to assess the significance of differences.

**Figure S5. Clustering analysis of the protein sequence characteristics of all 261 predicted LNM BGCs from the UHGG and NCBI Paenibacillus genomes using BiG-SCAPE.**
